# New Perspectives on BolA: A Still Mysterious Protein Connecting Morphogenesis, Biofilm Production, Virulence, Iron Metabolism, and Stress Survival

**DOI:** 10.3390/microorganisms11030632

**Published:** 2023-03-01

**Authors:** Ana Alves da Silva, Lisete Galego, Cecília Maria Arraiano

**Affiliations:** Instituto de Tecnologia Química e Biológica António Xavier, Universidade Nova de Lisboa, Av. da República, 2780-157 Oeiras, Portugal

**Keywords:** *bolA*, BolA-like proteins, phosphorylation, biofilm, flagella, virulence, c-di-GMP, IbaG, glutaredoxin, Fe-S proteins

## Abstract

The BolA-like protein family is widespread among prokaryotes and eukaryotes. BolA was originally described in *E. coli* as a gene induced in the stationary phase and in stress conditions. The BolA overexpression makes cells spherical. It was characterized as a transcription factor modulating cellular processes such as cell permeability, biofilm production, motility, and flagella assembly. BolA is important in the switch between motile and sedentary lifestyles having connections with the signaling molecule c-di-GMP. BolA was considered a virulence factor in pathogens such as *Salmonella* Typhimurium and *Klebsiella pneumoniae* and it promotes bacterial survival when facing stresses due to host defenses. In *E. coli*, the BolA homologue IbaG is associated with resistance to acidic stress, and in *Vibrio cholerae*, IbaG is important for animal cell colonization. Recently, it was demonstrated that BolA is phosphorylated and this modification is important for the stability/turnover of BolA and its activity as a transcription factor. The results indicate that there is a physical interaction between BolA-like proteins and the CGFS-type Grx proteins during the biogenesis of Fe-S clusters, iron trafficking and storage. We also review recent progress regarding the cellular and molecular mechanisms by which BolA/Grx protein complexes are involved in the regulation of iron homeostasis in eukaryotes and prokaryotes.

## 1. BolA Function and Regulation

### 1.1. The Role of BolA in E. coli Survival

**Figure 1:**
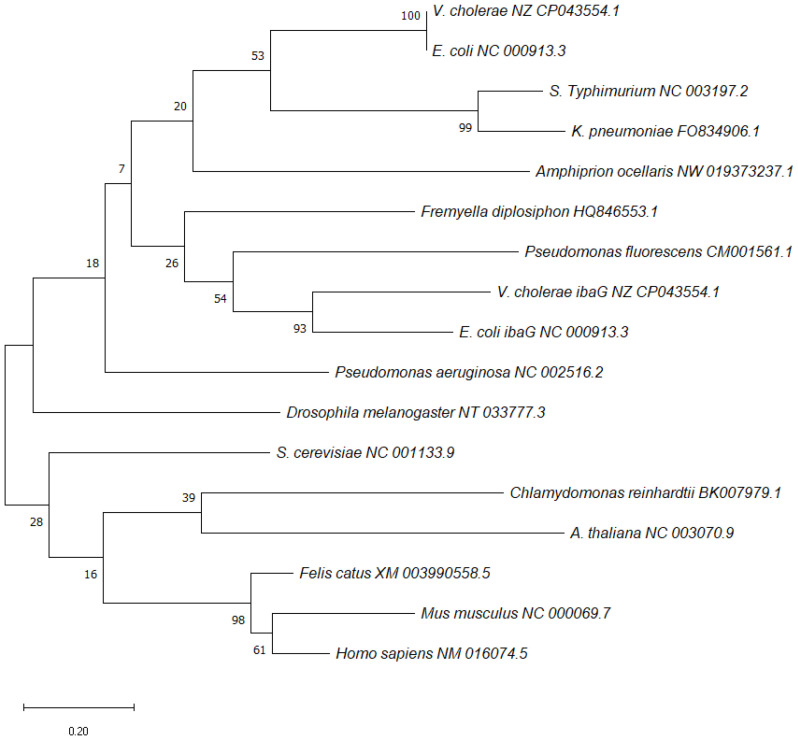
Maximum-likelihood phylogenetic reconstructions of *bolA1* using MEGA software v11.0.11 [13]. Distance estimation was obtained by Tamura–Nei model. Numbers at the nodes represent bootstrap values (%) based on 1000 replicates. Gene sequences were aligned using MUSCLE and the trees were reconstructed using default settings. The sequences of each gene were obtained from NCBI database [12] and the GenBank accession numbers are written next to each species.

BolA was discovered in *E. coli* in the 1980s and its name is due to its ability to produce osmotically stable spherical cells when overexpressed. It was shown to be involved in switching the cells between elongation and septation systems [1] during the cell division cycle, and the expression of *bolA* was shown to be growth-rate regulated, being induced during the transition into stationary phase [2,3,4]. BolA overexpression was responsible for spherical morphology in rod-shaped *E. coli* cells. Later, it was shown that BolA could also be induced in the exponential phase of growth, in response to several stresses [5,6,7]. BolA-like proteins constitute a widely conserved family of proteins widespread among prokaryotes and eukaryotes [1,8]. Phylogenetic analyses allowed to group BolA proteins into four subfamilies: BolA1-like (present in both prokaryotes and eukaryotes), BolA2-like and BolA3-like (found in eukaryotes) and BolA4-like (present only in photosynthetic organisms) [1,9,10,11]. A diversity of phenotypes has been linked to this protein family, although the molecular mechanisms that mediate BolA cellular effects are not yet well understood. Often, organisms encode several BolA members, performing different functions within a species and across the species. A phylogenetic tree for *bolA* was constructed based on the sequences of *bolA1* genes from different species (Figure 1). The sequences of each gene were obtained from the NCBI database [12]. The phylogenetic reconstruction was created in MEGA software v11.0.11 [13].

In *E. coli*, BolA is a small protein (≈12 kDa) [14] which is induced at the stationary phase of growth and by several stresses. It has been linked to membrane permeability, motility, cell morphology and biofilm development [1,4,5,6,7,15]. In 1988, Aldea and colleagues discovered that BolA is a *FtsZ*-dependent morphogene, and its overexpression made *E. coli* rod-shaped cells become spherical. This gave origin to the name of the gene: *bolA* (meaning ball) [1]. The mechanism by which BolA affects cell morphology is mediated by different factors. For instance, BolA binds to the promoter region of *mreBCD* decreasing the expression of the actin-like *mreB* [16]), and upregulates the genes *dacA* and *dacC* which codify the two main d-d-carboxypeptidases (respectively Penicillin-binding protein PBP5 and PBP6), regulating peptidoglycan biosynthesis [1,15,17]. It was also shown that BolA controlled the transcription of *ampC* (AmpC), a class C beta-lactamase, thus connecting for the first-time penicillin-binding proteins (PBPs) and beta-lactamases at the level of gene regulation [15].

The overexpression of BolA has an influence in the outer membrane permeability of *E. coli*. High levels of BolA have been shown to increase the ratio of OmpC/OmpF porins, turning the cell less permeable, and conferring protection from unfavorable environments [5]. Overexpression of BolA could even confer protection from detergents and from the antibiotic Vancomycin.

BolA is controlled at transcriptional, post-transcriptional and post-translational levels. Transcription of *bolA* can start at two different promoters. P2 is a constitutive promoter that is under the control of σ^70^ and is detectable in low amounts during all stages of growth. P1 is located 80 nt downstream, is under the control of σ^S^ and is expressed in stationary phase or under stress conditions [4,6]. For instance, in the face of stress conditions such as heat shock and acidic stress, *bolA1p* mRNA levels are increased [6]. Heat shock induction is almost immediate while the acidic stress is associated with a more gradual induction of *bolA* mRNA. In response to carbon starvation and osmotic shock *bolA1p* is highly induced and the level of its expression can largely exceed the ones reached in the stationary phase. These stresses make cells change their morphology to a rounder shape similar to those cells in which BolA is overexpressed in the stationary phase. On the other hand, oxidative stress leads to a moderate increase in mRNA *bolA1p* levels inhibiting growth and viability [6]. H-NS, a histone-like protein, was found to negatively regulate *bolA* expression in vivo and to interact with both *bolA1p* and *bolA2p* regions in vitro [18]. OmpR, in its phosphorylated form (phospho-OmpR), binds to the OmpR-binding region of *bolA1*, repressing its transcription [19]. Endoribonuclease RNase III acts as a post-transcriptional modulator of *bolA* expression under carbon-starvation conditions [20]. RNase III positively regulates *bolA1p* mRNA levels and stabilities. RNase III is furthermore shown to be necessary for the normal expression of σ^S^, ensuring normal levels of *rpoS* mRNA and σ^S^ protein under glucose starvation. Accordingly, under this stress, *bolA* transcript is increased and is more stable. This shows that *bolA* transcriptional and post-transcriptional controls are consonant to achieve the global regulation of the expression of this gene [5]. In 1997, Cao and Sarkar discovered that poly (A)-polymerase was able to directly regulate mRNA levels of both *bolA* and *rpoS*, and *bolA* transcript could be polyadenylated at its 3′end [20,21].

### 1.2. The Role of BolA in Virulence

Previous work from our lab indicated that BolA could be involved in different pathways directly related to bacterial virulence [22]. *Salmonella enterica* serovar Typhimurium (*S.* Typhimurium) is a pathogen that makes use of several virulence factors in order to overcome host defenses surviving inside host cells [23,24]. In order to unravel the role of BolA protein in the virulence of *S.* Typhimurium, the greater wax moth *Galleria mellonella*, was used as the infection model. *G. mellonella* has been extensively used as a model organism for a wide range of bacterial species including *S.* Typhimurium. BolA proved to be a determinant factor in the virulence capacity of *S.* Typhimurium and in its ability to survive and overcome host defenses. It conferred resistance to acidic and oxidative stress promoting its survival under harsh conditions [25]. When cells were infected with *S.* Typhimurium the wild-type bacteria could survive and multiply but the number of bacteria inside each cell was substantially reduced in the *S.* Typhimurium *bolA* deletion mutant.

To further explore the role of BolA in virulence, *S.* Typhimurium metabolism was investigated. Using 1H-NMR metabolomics, the metabolic differences between strains expressing different levels of BolA in a minimal virulence-inducing medium (LPM medium) were accessed. The strain overexpressing BolA revealed increased levels of acetate, valine, alanine, NAD+, succinate, coenzyme A, glutathione, and putrescine. These metabolites are implicated in pathways related to stress resistance and virulence. This suggests that BolA has an important role in metabolic regulation and that potentiates the virulence of *S.* Typhimurium [26].

Recently, BolA has also been identified as a virulence factor in *Klebsiella pneumoniae* [27] *K. pneumonia bolA* deletant mutants are less resistant to bile and oxidative stresses than wild-type cells. BolA is required for maintaining a proper cell morphology in the stationary phase of growth. In a *Galleria melonella* infection model, the larvae infected with *bolA* deletant *K. pneumoniae*, survived 53% more than the larvae infected with wild-type strain. BolA promoted the adhesion of *K. pneumoniae* to human cancer epithelial cells and significantly decreased the bacterial ability to colonize the liver, spleen, lung and kidney organs in a mouse model. Additionally, the formation of liver abscesses was not observed in mice infected with *bolA* deletant *K. pneumoniae*. BolA positively regulated siderophores production and biofilm formation as well as metabolites related to stress response and virulence (agmatine, cadaverine, guanosine, flavin adenine dinucleotide [FAD] and d-biotin). According to the authors, the downregulation of these metabolites may be the factor leading to the loss of virulence and stress resistance of the Δ*bolA* strain of *K. pneumoniae* [27].

## 2. BolA and Biofilms

BolA is known to be highly expressed in the stationary phase of bacteria growth and also in face of stress conditions. This suggests that BolA could be implicated in the formation of biofilms. It is estimated that biofilms occur in most parts of bacterial infections. These are consortia of microorganisms living in a reversible planktonic and sessile state, in which they aggregate to each other, sometimes attached to a surface. They are surrounded by a self-produced matrix composed mainly of proteins, DNA and polysaccharides. They differ phenotypically from planktonic cells in their gene transcription and growth rate [28,29]. Biofilms are associated with antibiotic resistance or tolerance, probably due to their organization that protects the bacteria present in the inner layers from the antimicrobial agents and by promoting the horizontal gene transfer of resistance genes. The role of BolA in biofilm formation was first reported by Vieira and colleagues in 2004. The authors presented evidence that the overexpression of BolA in *E. coli* promoted biofilm formation while the absence of the gene produced thinner biofilms. Moreover, they showed that, under stress conditions such as nutrient depletion or oxidative stress, the *bolA* mutant had a much lower biofilm production when compared to the wild-type strain [7]. The biofilm formation and adherence pattern of *E. coli* to different materials, such as silicone, stainless steel and polypropylene, was investigated and *bolA* mutants showed reduced biofilm thickness in silicone surfaces [30].

When switching from a planktonic to sessile lifestyle, motile bacteria are required to shut down their motility. It is now known that mutations in diverse regulatory genes have opposed effects in biofilm formation and in motility [31]. In 2015, Dressaire and colleagues demonstrated the importance of BolA in the switch between motile and sedentary life-styles in *E. coli*. BolA was shown to downregulate the master regulator of flagellar synthesis FlhDC and promote the production of curli, fimbriae and biofilm matrix. The overexpression of BolA significantly impaired bacterial swimming capacity. Recurring to immunofluorescence the authors demonstrated that, in the presence of higher levels of BolA, flagella could not be observed, suggesting that BolA is impairing flagellar biosynthesis or is interfering with their assembly [22].

Later on, Azam and colleagues deleted the *bolA* gene in *E. coli* using CRISPR interference [32] The authors found that it led to a significant reduction in the expression of curli amyloid’s genes *csgA* and *csgD*. Moreover, the expression of *fimH* gene, involved in fimbriae production, was also decreased, suggesting a role of BolA in the regulation of curli and fimbriae production. The knockout strain had a diminished biofilm formation as well as less curli and fimbriae. The extracellular DNA, proteins and sugars, which are important for the biofilm establishment, were significantly reduced in the *bolA* knockout mutant. This was previously shown when comparing wt with overexpression strain and *bolA* mutant [22]. Microscopy studies showed differences between *bolA* deletant and the wild-type strain. In absence of *bolA* cells exhibited a smoother cell surface, revealing a decreased curli and fimbriae presence [32]. The biofilm thickness was reduced as well as the cell aggregation, as had been previously shown by Vieira and colleagues in 2004 [7]. Together, these findings reinforce that BolA leads to the inhibition of biofilm formation through curli and fimbriae inhibition [7,22,32].

The bacterial second messenger c-di-GMP plays an important role in the transition from the motile to biofilm lifestyle. This messenger is synthesized by diguanylate cyclases (DGCs) leading to the production of adhesins and biofilm matrix components [33,34]. In turn, the hydrolyzation of c-di-GMP by phosphodiesterases (PDEs) promotes motility and the dispersion of biofilm cells [35,36,37]. Given that BolA and c-di-GMP share common features, such as being regulators of bacterial motility, Moreira and colleagues investigated their relationship. The authors showed that in a *bolA* deletion mutant, the c-di-GMP concentration in the cells was increased, while in an overexpressing BolA strain, it was reduced. BolA was found to be involved in the transcriptional regulation of diguanylate cyclases and phosphodiesterases through the direct binding of BolA to the promotor region of these genes. Moreover, c-di-GMP can regulate *bolA* expression as proven by the decreased *bolA* mRNA levels in presence of high levels of c-di-GMP. The authors proposed the existence of a negative-feedback modulation between *bolA* and c-di-GMP that leads to a proper physiological response of the cell, crucial for the transition between sessile and motile lifestyles [38] However, it is quite interesting to note that, even with high levels of c-di-GMP, there was no production of biofilm in the *bolA* deletion mutant. Galego and colleagues (2021) have also proven that in vivo phosphorylation of BolA was required for its role in biofilm formation (see next chapter). The authors suggested that it might be due to the loss of capacity of BolA to regulate target genes involved in biofilm formation [14].

## 3. BolA Phosphorylation and Interaction with Other Cellular Proteins

Protein phosphorylation can play a crucial role in cellular physiology, metabolic pathways, gene expression and virulence. This modification can affect protein activity, folding, stability and interaction with nucleic acids or other proteins [39,40]. The phosphorylation *status* of *E. coli* BolA protein was analyzed by Galego et al. (2021). They observed that BolA is phosphorylated in vivo in four highly conserved residues: two serine’s and a threonine located in the predicted HTH motif involved in DNA binding, and a serine located at the C-terminal non-structured region of the coding sequence. They have done a systematic site replacement of Ser/Thr to Ala/Lys as non-phosphorylated residues. Using single and combined double, triple and quadruple phosphor-mutants, they have investigated the outcome of these phosphosites on the stability, folding and activity of *E. coli* BolA protein. They observed that the dephosphorylation of these residues affects the stability/turnover of BolA protein and the morphology of the cells. Microscopic observation revealed that overexpression of BolA cannot produce round-shaped cells if BolA is Ser/Thr dephosphorylated in the HTH motif of the gene, while the overexpression of WT BolA strain produced round-shaped cells. Dephosphorylated BolA at the serine located in the C-terminal region of the protein produces larger olive/round-shaped cells [14]. In addition, the stability of BolA protein is higher in dephosphorylated C-terminal serine mutants and it is lower if the dephosphorylated residues are located in the DNA-binding motif. The correct expression and polymerization of actin-like MreB protein are essential for bacterial cell cytoskeleton and maintenance of cellular rod-shape [41]. *E. coli* has only a single MreB homolog [42] and MreB depletion results in the loss of the rod-like shape producing a round phenotype [43]. It was observed that BolA can reduce the expression of *mreB* [16] and later, by ChiP Seq, it was shown that BolA can directly bind *mreB* promoter [22]. Overexpression of the BolA protein disrupted the spatial organization of the cytoskeleton by downregulation of *mreB* expression, inducing a spherical-shaped morphology [6,16]. Galego et al. (2021) demonstrated that dephosphorylation of BolA has a highly reduced binding affinity to the *mreB* promoter, affecting negatively its capacity to regulate *mreB* expression. They performed *bolA* and *mreB* semi-quantitative RT-PCR, using overexpressing wild-type cells and BolA phospho-mutants, in the late stages of growth. They demonstrated that expression of *bolA* mRNA is similar in the wild type and in all phospho-mutant strains, while the expression of *mreB* was lower in the BolA wild-type strain. Western blot analysis, probed with MreB antibody, also revealed that expression of MreB was lower in wild-type overexpressing BolA than in overexpressing BolA phospho-mutant strains. These results demonstrated that in vivo phosphorylation of *E. coli* BolA is an important event in conferring its capacity to act as a transcription factor.

As already referred, one of the most remarkable functions associated with BolA, is its involvement in biofilm formation. Galego and colleges have investigated the impact of phosphorylation on the capacity of BolA to induce biofilm. They observed that, when BolA is dephosphorylated in the DNA binding motif, *E. coli* cells display a strong decrease in biofilm development when compared to the wild type, probably due to the lower capacity to regulate target genes involved in biofilm formation. Accordingly, they concluded that in vivo phosphorylation of BolA plays a role in the modulation of its activity as a transcription factor, as a regulator of cell morphology, and in biofilm development [14].

**Figure 2:**
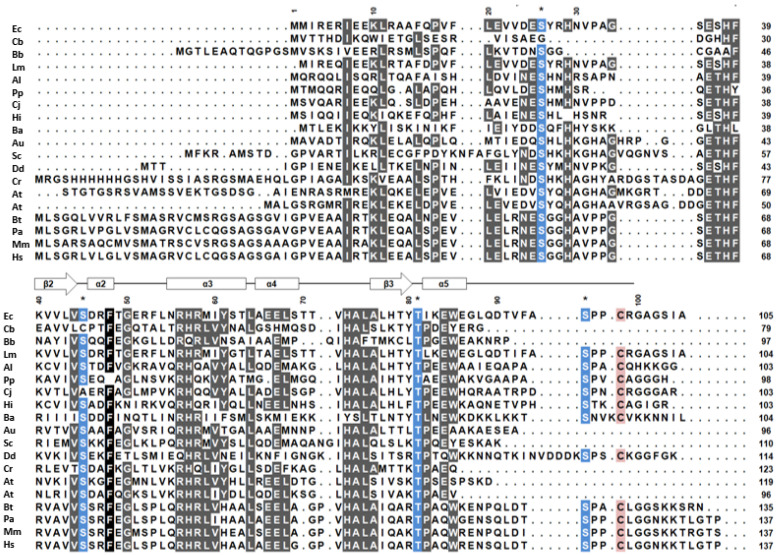
Phylogenetic analysis of BolA-like proteins. *ClustalW* multiple sequence alignment of *E. coli* BolA protein, used as template, with BolA-like proteins from prokaryotes and some eukaryotic organisms. Identical residues are in gray and black. The putative *E. coli* phosphorylated residues, are highly conserved and colored in blue. The only cysteine present in *E. coli* BolA is also highly conserved and is colored in rose. The * represents the phosphorylated sites in *E. coli*. The secondary αββαβ structure was predicted in Net Secondary Structure Prediction Jalview. The secondary structure, regarding *E. coli* BolA protein, is shown above the alignment. The sequence ID for the proteins is presented: Ec: *E. coli*-WP_024139872.1; Cb: *Coxiella burnetii*- AAO90126.1; Bv: *Babesia bovis*-XP_001609842.1; Lm: *Listeria monocytogenes*-WP_024139872.1; Al: *Agitococcus lubricus*-WP_107866244.1; Pp: *Pseudomonas putida*-WP_132844560.1; Cj: *Chromohalobacter japonicus*-WP_040243100.1; Hi: *Haemophilus influenzae*-WP_015701425.1; Ba: *Buchnera aphidicola*-WP_009874425.1; Au: *Aerococcus urinae*WP_111852928.1; Sc: *Saccharomyces cerevisiae*-NP_075206.1; Dd: *Dictyostelium discoideum*XP_644296.1; Cr: *Chlamydomonas reinhardtii*- XP_001702905.1; At: *Arabidopsis thaliana* 1-BAD43461.1; At: *Arabidopsis thaliana* 2-Q84W65; Bt: *Bos taurus*-NP_001029524.1; Pa: *Pongo abelli* NP_001125298.1; Mm: *Mus musculus*-NP_081251.1; Hs: *Homo sapiens*-NP_001307954.1 [45,46].

Most BolA-like proteins range in length from 76 to 123 amino acid residues [44]. Comparative sequence analysis revealed close homology of *E. coli* BolA protein to BolA-like proteins from other prokaryotes and most eukaryotes including *Saccharomyces cerevisiae*, *Arabidopsis thaliana* or *Homo sapiens* (see Figure 2).

The analysis of this sequence alignment revealed that several residues are highly conserved from bacteria to *Homo sapiens*. A motif of SxxF(x18)E(x5)H was conserved in all BolA-like proteins from prokaryotes to eukaryotes including plants and mammalians. The histidine residue, in this highly conserved motif, is likely required to covalently chelate an iron of the FeS cluster.

**Figure 3:**
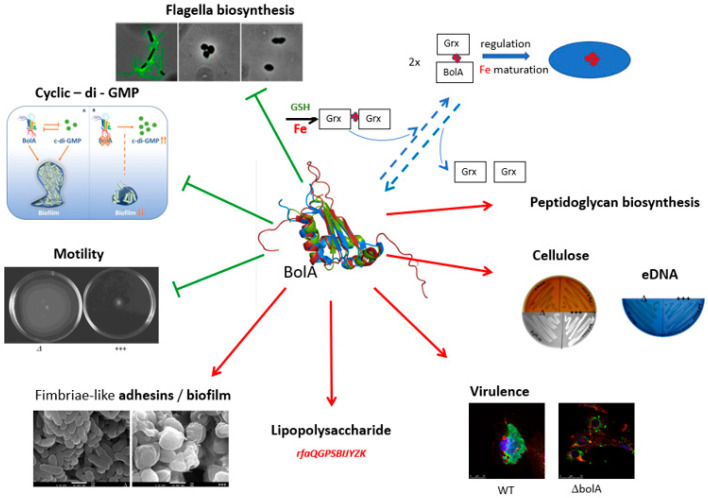
Pleiotropic effects of *E. coli* BolA. In green are represented features/mechanisms repressed by BolA. Mechanisms promoted by BolA can be seen in red. Blue arrows represent the role of BolA in iron homeostasis, a pathway that is still not well understood. The cartoon in the center represents superposition of BolA NMR structure of three BolA proteins (B): *E. coli* (PDB ID 2DHM, red): *C. burnetti* (PDB ID 3TR3, green), *B. bovis* (PDB ID 3O2E, blue) [14].

NMR structure of mouse BolA1 (PDB ID 1V60) and *E. coli* BolA (PBD ID 2 DHM) revealed similarities to nucleic acid-binding proteins [11] and display a helix-turn-helix motif DNA-binding motif (αββαβ structure, Figure 2) [11]. This motif may correspond to a DNA-binding domain involved in the regulation of different genes [11,14,22]. The cartoon, in the center of Figure 3, represents the superposition of BolA NMR structure of three BolA proteins *E. coli* (PDB ID 2DHM, red): *C. burnetti* (PDB ID 3TR3, green), *B. bovis* (PDB ID 3O2E, blue) [14]. This structure superposition revealed that the overall structure of *E. coli* BolA is similar to other BolA-like proteins and that the structural positions of the phosphorylated residues are conserved.

## 4. BolA Interacts with Grx Mediating Intracellular Iron Homeostasis

Data from bioinformatics approaches, affinity protein purification and protein–protein interactions indicate a physical link between BolA-like proteins and the monothiol glutaredoxin (Grx) proteins suggesting a functional interaction between these two protein families [9,47,48]. Many cellular reactions require metalloproteins as cofactors in enzymatic reactions, namely iron (Fe) and iron-sulfur (Fe-S) clusters. BolA and Grx proteins often cluster with Fe-S biogenesis operon supporting a strong phylogenetic connection between these two proteins families in iron homeostasis [45,49,50]. Organisms must strictly regulate iron uptake and usage to maintain optimal intracellular levels. Low intracellular levels disrupt the activity of iron-dependent enzymes and iron overdose can prevent iron trafficking, disturbing the normal redox state of the cell [51,52].

Monothiol Grx proteins perform a role in iron metabolism participating in iron storage, iron sensing, coordination and transport of Fe-S [53]. This protein family contains a CGFS motif as an active site, which is involved in the coordination of the [2Fe-2S] cluster, via the cysteine active site and the sulfhydryl residue of a molecule of glutathione-S-transferase (GSH), which is non-covalently bound adjacent to the Grx motif site. GSH is a ubiquitous nucleophile required for the redox homeostasis, detoxification and synthesis/maturation of Fe clusters proteins [54,55,56,57,58].

CGFS-type monothiol Grx proteins are classified into two groups: single-domain CGFS Grxs and multi-domain CGFS Grxs. Single-domain CGFS Grxs are found in prokaryote and eukaryote organelles (mitochondria and chloroplasts) and have been implicated in the redox regulation of Fe-S cluster proteins [56,59,60]. Multi-domain CGFS Grxs are exclusive for eukaryotes with cytosolic/nuclear localization where they play a role in the regulation of Fe trafficking and homeostasis and in the maturation of Fe-S proteins owing to their capacity to ligate Fe-S clusters with their protein partners [55,61,62]. Single- or multiple-domain CGFS Grxs proteins form [2Fe-2S]-bridged homodimers, via the cysteine motif active site, and two GSH molecules, which leads to dimerization of monothiol Grxs proteins [53,55,63].

The first evidence about the physical interaction between monothiol CGFS-type Grxs and BolA-like proteins in the biogenesis of Fe-S proteins was obtained from *Saccharomyces cerevisiae* [64,65]. Fungi express three CGFS-type Grxs homologues (Grx3, Grx4 and Grx5) and three non-redundant BolA proteins paralogs (BolA1, BolA2 and BolA3). Yeast BolA2 protein is localized in the cytosol/nucleus while BolA1/BolA3 (mBols) are expressed in the mitochondria. The CGFS-type monothiol Grx5 is localized in yeast mitochondria where it facilitates the transfer of nascent Fe-S clusters to target proteins. Its absence prevents respiratory growth and causes the accumulation of free iron in the cell [45,66]. The molecular function of the yeast BolAs (BolA1 and BolA3), and their interaction with the CGFS Grx5 protein, were largely studied by Uzarska and coworkers [67]. They characterized BolA1 and BolA3 proteins as specific mitochondrial ISC (iron-sulfur cluster) assembly factors that facilitate [4Fe-4S] cluster insertion into a subset of mitochondrial Fe-S acceptor proteins such as lipoate synthase and succinate dehydrogenase. The ISC biosynthesis pathway is found in many bacteria and in the mitochondria of eukaryotes and are protein cofactors involved in oxidative respiration, photosynthesis, nitrogen fixation and DNA replication/repairs [68]. Mutants for both of these two BolA proteins did not properly perform the assembly of some Fe-S proteins namely lipoic acid synthase. They demonstrated that BolA1 and BolA3 perform the overlapping function in the formation of dimeric complexes with both mitochondrial monothiol Grx5 and Nfu1 (Nitrogen-Fixation-Subunit-U) proteins playing a critical role at the late stage of Fe-s cluster insertion into the target proteins [67].

In *S. cerevisiae*, the transcription factor Aft1 (Fe-responsive transcription factor) controls genes involved in iron uptake and storage. Under high cellular iron conditions, this protein is localized in the cytosol and moves to the nucleus under reduced iron conditions, activating the transcription of the genes involved in iron uptake [69,70]. The yeast cytosolic BolA2-like protein, formerly termed Fra2 (Fe Repressor of Activation), and its CGFS-type monothiol Grx3- and Grx4-binding partners, have a role in the regulation of iron homeostasis by mediation the regulation of Aft1/Aft2 [65,71]. A deletion mutant for BolA-like protein in *S. cerevisiae* exhibited phenotypes suggestive of the misregulation of iron metabolism including accumulation of mitochondrial iron [71].

**Figure 4:**
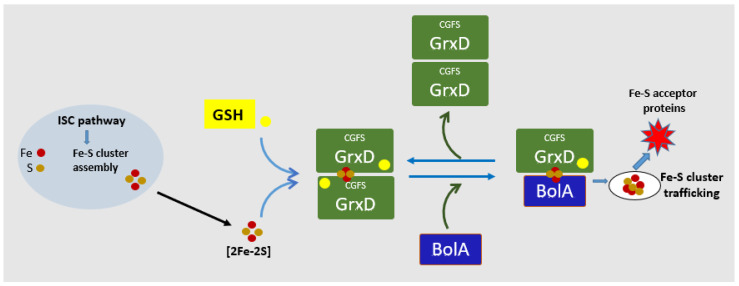
Schematic diagram of FeS cluster transfer/maturation in *E. coli*. In *E. coli* the synthesis of FeS clusters is catalyzed by complex machinery that includes ISC system. [2Fe-2S] clusters are distributed in a process that depends on the Glutathione-S-transferase (GSH, colored in yellow), CGFS-type GrxD homodimers (colored in green) and GrxD-BolA heterocomplexes, (BolA colored in blue). The reactions are reversible and monomeric BolAs or GrxD-BolA could be reformed. The physiological relevance of each form has not been elucidated but this interconversion could play a role in FeS trafficking and homeostasis.

Similar genetic, biochemical and biophysical studies performed in *E. coli*, *Arabidopsis* and *Homo sapiens*, with homologues for CGFS-type Grxs and BolA-like proteins, confirmed the results obtained in yeast. *E. coli* genome encodes only one CGFS-type monothiol glutaredoxin (Grx4 or GrxD), with high sequence homology to yeast Grx5, containing a single CGFS Grx domain [72]. This Grx4/D protein is highly expressed in *E. coli*, at the stationary phase of growth, and is induced under stress conditions, namely iron starvation. Grx4/D deletion mutant is sensitive to iron depletion and reveals synthetic lethality in combination with a mutation in several genes in *isc* operon (iron-sulfur cluster) suggesting the Grx4/D function in a parallel pathway for Fe-S biogenesis [47,73,74]. It was demonstrated that CGFS GrxD co-purifies with the affinity purified *E. coli* BolA and forms both homodimer and heterodimers (with BolA) Fe-S cluster containing complexes [47]. It was suggested that the shift of GrxD from a dimeric to the monomeric state is coupled with the release of the Fe-S cluster, representing a way by which GrxD may transfer the Fe-S cluster to target proteins [61,74]. A schematic model of the formation of the BolA/Grx complex in *E. coli* and FeS transfer is shown in Figure 4. As observed in yeast, heterodimer GrxD-BolA complexes display increased stability over their homodimer counterparts. However, the GrxD homodimer is a better Fe-S cluster donner than the GrxD-BolA heterodimer. Homodimers transfer their Fe-S clusters to apo-ferredoxin in vitro more efficiently which suggests different functional roles for the [2Fe-2S]-bridged homo and heterodimeric complex in vivo [47,55]. Indeed, 3D structures comparison of the monomer and dimer GrxD have shown some functionally conformational change [61]. Since both Grx and BolA are highly induced at a stationary phase of growth and form heterodimer complexes Yeung, N. and coworkers (2011) suggested that BolA might be the factor that regulates these conformational changes. BolA may promote the conversion of a GrxD homodimer into a GrxD-BolA heterodimer complex limiting the activity of the GrxD homodimer and increasing the activity of the GrxD-BolA heterodimeric complex [47].

Genetic and biochemical analysis of the *E. coli* IbaG protein, the other *E. coli* BolA-like protein, reveal that mutations in *Grx4/D* or *IbaG* genes increase the growth defects of strains mutated in the *isc* operon suggesting that Grx4 and IbaG proteins mediate a process of iron-sulfur cluster assembly [48]. By in vitro studies, Dlouhy and coworkers demonstrated that *E. coli* IbaG binds the Grx4 homodimer to form Grx4-IbaG heterodimers in the apo form as well as the holo form with a [2Fe-2S]-cluster bridging the two proteins. Mutagenesis and spectroscopic studies of the Grx4-IbaG complex indicate that a conserved His63 from IbaG ligates the Fe-S cluster. Substitution of this His for Ala or Cys favours the formation of [2Fe-2S] Grx4 homodimer and raises the GSH content of the complex. Thus, in the absence of His63, a cluster coordination site with Cys ligands, similar to the homodimer, is favored over the heterodimer formation [48]. These results suggest that IbaG and Grx4 complexes may function as an alternative pathway to Fe-S cluster assembly and trafficking in *E. coli*, although it is not known if the IbaG and BolA interactions with Grx4/D were performed similarly.

A physical interaction was also described between some CGFS-type Grxs and BolA-like proteins in *Arabidopsis thaliana* and *Human sapiens* suggesting that Grx-BolA interaction is a universal cellular event [9,10,45]. Specific iron metabolism systems exist for the mitochondrial/chloroplast and for the cytosolic/nuclear compartments that selectively distribute Fe and Fe-S in the subcellular compartment. Specific BolA-like proteins, expressed in several subcellular compartments, may have different effects in the Fe-S biosynthesis/trafficking system. Distinct CGFS-type monothiol Grxs are expressed in the mitochondria/chloroplast and cytosol. Cytosolic CGFS-type monothiol Grxs differ from their mitochondrial/chloroplast paralogs in that they contain an amino-terminal thioredoxin (Trx)-like domain, followed by one or more Grxs domains [75]. Genetic evidence suggests that mitochondrial Grxs are involved in the transfer of newly assembled Fe-S clusters to recipient apoproteins whereas cytosolic monothiol CGFS-type Grxs are thought to play a role in the redox homeostasis [62,67,72,76].

In *Arabidopsis thaliana*, the BolA family contains four members including the sulfotransferase SufE1, which contains a C-terminal BolA domain [9,77]. At least one BolA isoform is expressed in each *A. thaliana* subcellular compartment. BolA1 is plastidial, BolA2 and BolA3 are nucleus–cytoplasmic and BolA4 is plastidial and mitochondrial. Binary yeast two-hybrid experiments, confirmed by bimolecular fluorescence complementation, demonstrated that all *A. thaliana* BolAs and SufE1 (via its conserved reactive cysteine), can interact with all CGFS-type monothiol Grxs of the same subcellular compartment and this interaction may allow the activity of BolA protein to be redox-regulated by its Grx partner [78,79] Using three-dimensional modeling and other physical assays, Roret and colleagues characterized in *A. thaliana* the apo- and holo-heterodimers formed between AtBolAs and a monothiol AtGrxs. They showed that the AtBolA protein could interact with DNA and Grx apoforms using the same interaction surface. They suggested that the HTH DNA-binding motif of BolA may be also involved in glutaredoxin recognition and the formation of Grx-BolA apo-heterodimer may modify the nucleic acid binding capacity of BolA [78].

The *A. thaliana* AtBolA3 has a high similarity to the human BOLA3, it is located in the cytosol and is highly expressed in the roots [9]. Mutants for this BolA were more resistant to excess iron and oxidative stress. Under stress conditions, they have higher Fe-S protein activities and accumulated more iron in the root. In vivo experiments suggested that this *Arabidopsis* AtBolA3 interacts with the cytosolic AtGrx17 protein acting in roots, and has a suppressive role in the tolerance to an excess of iron and in oxidative stress [9].

In mammalian cells, the function of CGFS-type monothiol glutaredoxins (GLRXs) in iron metabolism is closely linked with the BOLA protein family. As demonstrated in yeast, humans express three BOLA members that can be identified by the presence of conserved sequence elements [67]. Human BOLA1 and BOLA3 are mitochondrial, with a specific role in the mitochondrial assembly pathway of iron-sulfur proteins and in the maturation of Fe-S clusters, and BOLA 2 is cytosolic, but without a well-defined physiological role [45,55,67,80]. Human BOLA1 is an orthologue of *S. pombe* Uvi31, which are evolutionary near to *E. coli* BolA, and human BOLA2 is an orthologue of *S. cerevisiae* Fra2. The physiological role of human BOLA3 has been better characterized in patients with Multiple Mitochondrial Dysfunction Syndrome (MMDS), a severe autosomal recessive disease caused by BOLA3 mutations. MMDS patients reveal a phenotype identical to patients mutated in the ISC factor with reduced energy production in mitochondria, decreased function of the respiratory chain complexes (I and II) and the lipoic acid-dependent enzymes [67,81,82,83]. This syndrome provides solid evidence for a direct role of *Homo sapiens* BOLA3 in mitochondrial Fe-S protein biogenesis as it was also reported in *S. cerevisiae* [81,84].

The results presented by Willems and coworkers suggest that the human BOLA1 performs a role in mitochondrial morphology and thiol redox potential regulation. The authors suggest that BOLA1 prevents mitochondrial morphology aberrations induced by GSH decrease and reduces the associated oxidative shift of the mitochondrial thiol redox potential [85].

Other studies, connecting human iron metabolism with CGFS GLRXs and BOLA, came from the interaction between GLRX3 and BOLA2. In cultured human cells, it was demonstrated that the cytosolic GLRX3/BolA2 complex represents a reservoir of [2Fe-2S], with the transfer of Fe-S via direct protein–protein interactions, which is compatible with a role of [2Fe-2S] cluster chaperone [66]. Frey and coworkers observed that mutations in the GLRX3 residues, required for the Fe-S cluster in vitro binding, prevent the binding of BOLA2 to GLRX3. Furthermore, cellular iron removal disrupts GLRX3/BOLA2 formation, whereas iron supplements increase its formation. Cellular GLRX3/BOL2 complexes increase 6–8-fold in response to increased iron forming a reservoir of Fe-S clusters. The complex facilitates Fe-S incorporation into CIAPIN 1 (essential component of the cytosolic Fe-S assembly system), a [2Fe-2S] protein involved in the cytosolic Fe-S assembly pathway. Authors demonstrated that this complex formation is highly dependent on the coordination of bridging Fe-S clusters.

Many results demonstrated that BolA-like protein forms dynamic complexes with CGFS-type Grxs proteins and helps regulate iron metabolism in both prokaryotes and eukaryotes; however, it is still lacking the full comprehension of the specific function of BolA in these regulatory pathways.

## 5. BolA Orthologues

The IbaG is the other *E. coli* BolA-like protein. It is induced by acid stress and it only shares 22% sequence identity and 36% sequence similarity with *E. coli* BolA [86]. The *ibaG* mRNA levels increase in response to acid stress and its deletion increases the sensitivity of the bacteria to acidic stress. *E. coli* *ibaG* and *bolA* single and double mutants grow faster and have higher viability in rich media when compared to the wild-type strain. Nevertheless, in the late stationary phase of growth, the deletion strains present lower viability pointing to their role in bacterial survival in harsh conditions. Contrary to BolA, the overexpression of IbaG does not impact the cell shape but can be deleterious to bacterial growth [86]. *IbaG* is transcribed in an operon together with *murA*, a gene involved in the synthesis of peptidoglycan precursors, suggesting that it may have a function in cell wall biosynthesis [86].

The characterization of the IbaG protein from *Vibrio cholerae* revealed that it also contributes to the biogenesis and maintenance of the cell envelope in this bacterium. In the exponential phase, *V. cholerae* deleted for the *IbaG* gene presented multipolar elongated and wider cells with an aberrant shape. This was not observed in the stationary phase. The mutant contained reduced amounts of peptidoglycan and lipopolysaccharide (LPS) and altered lipid profile. These mutants revealed an impaired capacity to colonize the infected animal intestine and have elevated sensitivity to antibiotics and detergents [86,87].

The characterization of a BolA homologue from *Pseudomonas fluorescens* showed that the mutant strain exhibited a smaller cell size upon carbon starvation and grew slower in a minimal medium with L-serine as the unique nitrogen source [88]. Contrary to what is described for *E. coli*, *rpoS* did not significantly affect the expression of this *P. fluorescens bolA*. This gene in encoded in an operon where the two next ORFs encode proteins with homology to sulphurtransferases and protein disulphide isomerases. The successful complementation of the mutant strain with a plasmid-encoding *bolA* was only achieved when these two proteins were also present in the same plasmid. Results suggested that this *bolA* has different roles [88].

In the yeast *Schizosaccharomyces pombe*, the BolA orthologue *uvi31+* is an UV-inducible gene with a role in the cell cycle [89]. Increased expression of UVI*31+* protein accelerates spore germination, decreases proliferation rate, enhances cell size, controls the correct septum formation and cytokinesis, confers UV resistance and controls cell division [90]. In the algae *Chlamydomonas reinhardtii* there are five putative *bolA*-like genes. The *C. reinhardtii* BolA protein, with the highest homology to the *E. coli* BolA, was cloned and expressed in *E. coli*. This algal BolA protein reduced the size and promoted biofilm formation of the recombinant bacteria just like the overexpression of endogenous *E. coli* BolA [91]. This shows that heterologous expression of the *C. reinhardtii* BolA protein could successfully mimic what are the phenotypes of *E. coli* BolA, showing that these proteins are quite close in order to be able to perform this intra-kingdom complementation.

*Fremyella diplosiphon* is a freshwater cyanobacterium whose cells are rectangular under green light and smaller and spherical under red light. The role of BolA in the regulation of *F. diplosiphon* cell shape was investigated and it was proposed to be an essential gene as its complete deletion from the genome was not possible [92]. However, in a partially segregated Δ*bolA* strain, *F. diplosiphon* exhibited slower growth, some morphological defects, and accumulation of high levels of reactive oxygen species. In a wild-type strain, a higher expression of *bolA* was recorded under red light and this was correlated with a lower expression of *mreB* and *mreC*. In a strain, lacking the red/green responsive photoreceptor (Δ*rcaE*), the expression of *bolA* was altered under both lights. Results suggested that the expression of *mreB* and *mreC* is controlled by RcaE-dependent photoregulation of *bolA* expression establishing a role for BolA in *F. diplosiphon* cell morphology [44].

## 6. Conclusions

BolA was initially described as a stationary phase stress-responsive gene whose overexpression induced spherical shape in *E. coli*. Later, it was shown that it could be important for stress endurance and survival and could act be a transcription factor determinant in the switch between planktonic and sessil lifestyles, very relevant for biofilm formation. Subsequently, it was observed that BolA-like proteins constitute a widely conserved family in nature in all kingdoms of life. The role of BolA in bacterial virulence has been recognized in different species such as *S.* Typhimurium and *K. pneumoniae* where it has been shown to be important for successful host infection. One of the most notable features of BolA is its impact in biofilm formation. BolA downregulates the master regulator of flagellar synthesis FlhDC in *S.* Typhimurium and it promotes the production of curli, fimbriae and biofilm in *S.* Typhimurium and *E. coli*. A cross-talk with negative feedback between BolA and c-di-GMP, an important signaling molecule, was reported in *E. coli*.

It has been shown that the expression of *bolA* is highly regulated, at the transcriptional and post-transcriptional levels. Recently, it was also discovered that the BolA protein is phosphorylated. This post-translational modification was shown to be important for the stability/turnover of the protein and its activity as a transcription factor, acting in morphogenesis and biofilm formation. Several BolA homologues were studied, and it is known that their function can be different. The gene *ibaG*, is a *bolA* homologue in *E. coli* and confers resistance to acidic stress, but in *V. cholera* it is associated with cell morphology.

In the past decade, there has been some progress in the characterization of the ubiquitous monothiol CGFS-type Grx and GSH proteins in iron metabolism, particularly with the synthesis and maturation of Fe-S proteins. Organisms must strictly regulate iron uptake and usage to maintain optimal intracellular levels. *Grx* gene was implicated in a variety of distinct processes such as the biodegradation of xenobiotics, protection against chemical and oxidative stresses and antimicrobial drug resistance [92,93]. A link between the regulation of iron homeostasis and the response to oxidative stress, with increased intracellular iron, was shown to be correlated with cellular oxidative stress [92,93]. Recently, Grx and BolA-like proteins have emerged as a class of Fe-s cluster binding regulatory complexes. Both proteins are conserved in prokaryotes and eukaryotes so it is likely that Fe-S binding interaction between Grx and BolA is conserved through evolution. In *E. coli*, yeast, *Arabidopsis* and human cells it was demonstrated that BolA-like proteins form |2Fe-2S|-bridged heterodimers with Grx proteins that play a key role in signalling intracellular iron availability. They are proposed to facilitate the transfer of Fe-S clusters to target proteins during the maturation of Fe-S cluster-containing proteins. However, the interaction and physiological function of the complex GRx/BolA and the mechanisms involved in FeS transfer in the cell remains to be clarified. Identification of direct and indirect targets of both Grxs and BolA should allow a better characterization of proteins involved in the Fe-S cluster trafficking and metabolism. The combination of genetic approaches, biochemical and biophysical studies and proteomics analysis are required to characterize the specific molecular function of BolA protein in the Grx/BolA complex and their role in the maintenance of Fe homeostasis, protection against stresses, antimicrobial activity and biofilm development.

## Data Availability

Not applicable.
